# Implications of Transglutaminase-Mediated Protein Serotonylation in the Epigenetic Landscape, Small Cell Lung Cancer, and Beyond

**DOI:** 10.3390/cancers15041332

**Published:** 2023-02-20

**Authors:** Jason Lin, Shang-Chuen Wu

**Affiliations:** 1 Laboratory of Clinical Genomics, Chiba Cancer Center Research Institute, Chiba Cancer Center, Chiba 260-8717, Japan; 2 Division of Cell Therapy, Chiba Cancer Center Research Institute, Chiba Cancer Center, Chiba 260-8717, Japan; 3 Joint Program in Transfusion Medicine, Department of Pathology, Brigham and Women’s Hospital, Harvard Medical School, Boston, MA 02115, USA

**Keywords:** serotonylation, epigenomics, histone deacetylase inhibitor

## Abstract

**Simple Summary:**

From the landmark report of protein serotonylation on small GTPases in 2003 to the most recent discovery of serotonin-modified histone H3 leading to epigenetic changes, over the course of the past 20 years, there is still a cloud of mystery surrounding this rare post-translational modification, other than the fact that tissue transglutaminase, perhaps most famously known for its role in celiac disease, is the enzyme responsible for catalyzing this transamidation reaction. This review seeks to interpret the role of protein serotonylation in transcriptional regulation through some of the mechanistic and intertwining details of the modification, its potential modulation of the epigenetic landscape, as well as potential implications in lung and other types of cancer.

**Abstract:**

In the case of small-cell lung carcinoma, the highly metastatic nature of the disease and the propensity for several chromatin modifiers to harbor mutations suggest that epigenetic manipulation may also be a promising route for oncotherapy, but histone deacetylase inhibitors on their own do not appear to be particularly effective, suggesting that there may be other regulatory parameters that dictate the effectiveness of vorinostat’s reversal of histone deacetylation. Recent discoveries that serotonylation of histone H3 alters the permissibility of gene expression have led to renewed attention to this rare modification, as facilitated by transglutaminase 2, and at the same time introduce new questions about whether this modification belongs to a part of the concerted cohort of regulator events for modulating the epigenetic landscape. This review explores the mechanistic details behind protein serotonylation and its possible connections to the epigenome via histone modifications and glycan interactions and attempts to elucidate the role of transglutaminase 2, such that optimizations to existing histone deacetylase inhibitor designs or combination therapies may be devised for lung and other types of cancer.

## 1. Introduction

Lung cancer is often subdivided into small-cell lung cancer (SCLC) and non-small-cell lung cancer (NSCLC) and is generally found to be one of the leading causes of cancer-related deaths. In Japan, for instance, the 5-year survival rate for NSCLC has been estimated to be about 34.3%, while SCLC may be lower than 15.8% (Appendix A); prognosis tends to be poor regardless of the subtype. SCLC in particular can be highly metastatic and largely driven by a number of loss-of-function mutations found in tumor suppressor genes, for example, RB1 and p53 [1], that may lead to tumorigenesis becoming largely uncheckered. In certain SCLC cases, chromatin modifiers such as CREBBP, MLLs, and ARID1A/B carry deactivating mutations with severe implications [2,3], suggesting that the modulation of the SCLC epigenetic landscape may be an attractive therapeutic option. Histone deacetylases (HDACs), the enzymes capable of reversing lysine acetylation, in this regard, are potential cancer therapeutic targets, especially considering their frequent overexpression in cancer [4]. HDACs are either Zn^2+^ (classes I, II, and IV) or nicotinamide adenine dinucleotide-dependent (class III) [5], and while the contributions of specific HDAC subtypes to individual cancers have not been fully elucidated [5,6], HDAC inhibitor (HDI) candidates, for instance, suberoylanilide hydroxamic acid (SAHA, also known as vorinostat), can interfere with cancer progression. Vorinostat, for instance, enhanced cisplatin accessibility and increased histone H3 acetylation in SCLC cell lines [7]; the compound is similarly said to enhance gefitinib-induced cancer cell death in NSCLC [8] as well. Vorinostat is also a frequent subject in clinical trials, but the drug itself has not demonstrated sufficient efficacy as a treatment for either type of lung cancer (Appendix B). When administered as part of combination therapy with compounds such as carboplatin, brigatinib, erlotinib, pembrolizumab, etc., [7,8,9,10,11,12,13], vorinostat does show a certain level of effectiveness to suggest the presence of other regulatory parameters that can dictate the effectiveness of vorinostat’s reversal of histone deacetylation.

Recently, Farrelly and colleagues reported that histone H3 undergoes serotonylation at the Q5 residue [14] (Q5ser), and this modification sensitizes transcription factor II D binding. This finding led to renewed interest in protein serotonylation, a relatively uncommon post-translational modification (PTM) first reported by Walther et al. [15], as well as tissue transglutaminase (TGase2, TG2, or TGM2; TGM2 henceforth) and its peculiar ability to transamidate small biogenic amines onto glutamine residues. Protein serotonylation serves unique and interesting roles in the interplay of histone modifications, and to a further extent, the manipulation of the epigenetic landscape. Despite this discovery, however, there is still a knowledge gap in the phenomenon of protein serotonylation, from its intended purpose in signaling to its potential roles in epigenetic modulation. This review attempts to discuss the mechanism between protein serotonylation, as well as the potential implications of this modification in cancer and other diseases through its connections to epigenetics.

## 2. Transglutaminase 2 (TGM2)

**Figure 1:**
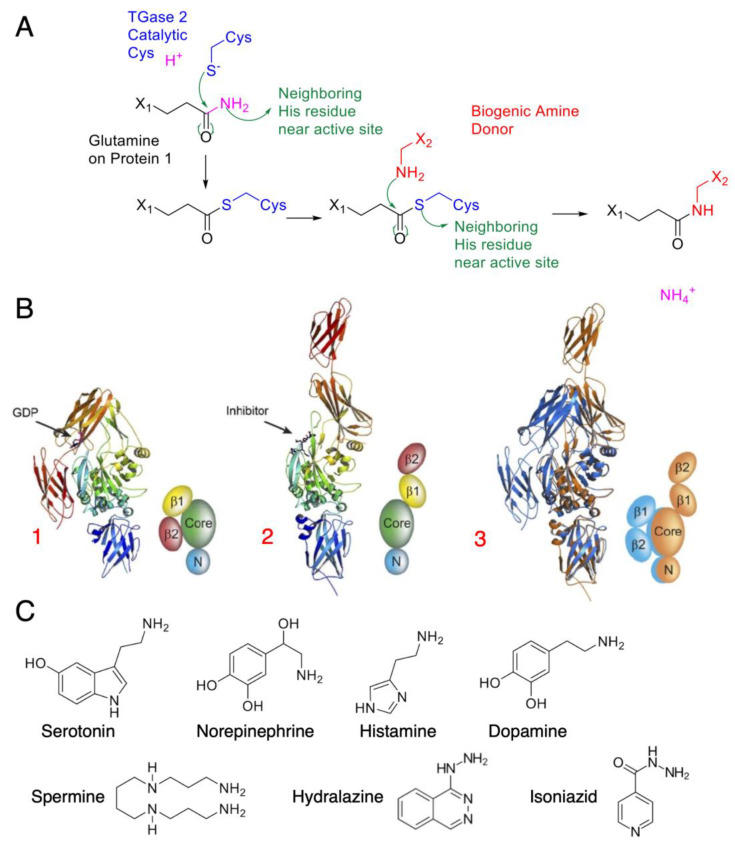
TGM2 mode of action and amine donor substrates: (**A**) Catalytic mechanism of TGM2, with green arrows indicating electron movement. TGM2 catalyzes transamidation reactions between a protein at its glutamine residue and various amine donors. The reaction is first initiated by a nucleophilic attack by the activated cysteine on the glutamine amide, forming the thioester intermediate. A biogenic amine donor leads to another nucleophilic attack and completes the transamidation reaction. (**B**) Crystal structure of tissue transglutaminase. TGM2 undergoes a large conformational change upon activation. In this figure, the crystal structures are shown as ribbons; simplified cartoons are included for clarity [16] (© 2007 Pinkas et al., distributed under the terms of the Creative Commons Attribution License, which permits unrestricted use, distribution, and reproduction in any medium, provided the original author and source are credited). The *N*-terminal β-sandwich is shown in blue (N), the catalytic domain (Core) in green, and the C-terminal β-barrels (β1 and β2) in yellow and red, respectively: (1) GDP-bound TGM2; (2) TGM2 inhibited with the active-site inhibitor Ac-P(DON)LPF-NH_2_; (3) the N-terminal β-sandwich and catalytic domains of the two structures are superimposed, highlighting the conformational change. The GDP-bound structure is shown in blue, and the inhibitor-bound structure is shown in gold. (**C**) Common amine substrates for TGM2, with each serving a different purpose as neurotransmitters and transduction agents.

Outside the public media’s recent fascination with glutens and celiac disease, TGM2 remains a relatively understudied enzyme, despite being one of the most ubiquitously distributed isozymes in the body. Human TGM2 consists of four domains: a fibronectin-binding domain and a catalytic domain, as well as two C-terminal β-barrels belonging to the fibronectin Type III CATH superfamily [16]. Upon activation, TGM2 may then crosslink protein glutamines with another amine donor in a reaction known as transamidation (Figure 1A). After the glutamine-containing amine acceptor binds to the enzyme, it forms a ɣ-glutamylthioester with the catalytic cysteine residue, leading to an acyl–enzyme intermediate. This linkage is accompanied by the release of ammonia. The amine donor proceeds to bind to the acyl–enzyme intermediate and attacks the thioester bond, subsequently restoring the catalytic cysteine to complete the transamidation reaction, with the formation of acyl–enzyme intermediate being rate-limiting [17]. Khosla and colleagues identified “open” and “closed” conformations spanning as distant as 120 angstroms (Figure 1B) upon calcium stimulation. A vicinal disulfide bond also exists between two surface cysteine residues, cysteines 370 and 371, contributing to the conformational change. TGM2 has a wide range of amine-donor specificity, from biogenic mono- and polyamines (Figure 1C) to other amine-containing compounds such as the antituberculosis agent isoniazid and the antihypertensive drug hydralazine [18].

**Figure 2:**
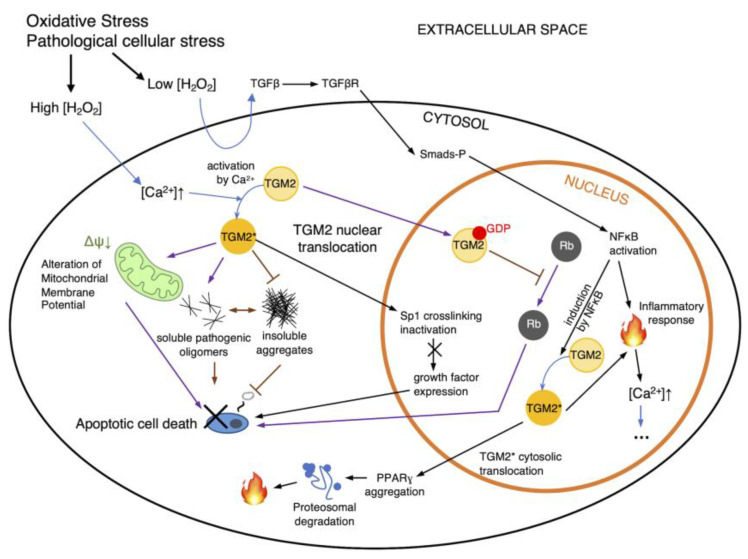
Regulation routes of TGM2. Intracellular ROS accumulation has a number of direct and indirect consequences on cell signaling pathways such as NFκB-TGM2 signaling (black arrows), leading to apoptosis or necrosis. Additionally, pathological cellular stresses can also affect TGM2’s transamidation activity and subsequently affect cell death. Additionally, cytosolic TGM2 may mediate cellular pathology by inhibiting the formation of insoluble protein aggregates and increasing levels of pathogenic soluble oligomers (purple arrows), or altering the mitochondrial membrane potential, all of which can lead to cell death. However, cell death may be ameliorated via the nuclear translocation of TGM2, which may trigger Rb interaction. NFκB may also induce TGM2 to generate an inflammatory response within the nucleus and in the cytosol, propagating and cascading intracellular calcium increase (indicated as ellipses). Black arrows indicate routes of TGM2 regulation as proposed by [22]; purple arrows indicate routes per [28]; H_2_O_2_, hydrogen peroxide; TGM2*, activated TGM2; blue arrows indicate state changes, such as the activation of TGM2.

TGM2 is mechanistically dependent on calcium regulation. The tight regulatory control of calcium uptake and release in theory ensures that TGM2 is also highly regulated except in the case of a significant interruption of redox homeostasis. The enzyme appears to have certain rules of specificity [19] but is relatively short-lived [20]. Increases in intracellular calcium concentration as a result of release from intracellular stores or ion transport from extracellular spaces [21], even in the absence of protein synthesis, can activate latent transglutaminase activity. The effects of redox regulation on TGM2 are via a number of different pathways [22] (Figure 2), with TGM2 activity likely to be modulated through cysteine oxidation in the form of cysteine *S*-thiolations, e.g., *S*-nitrosylation and *S*-glutathionylation. Experimental observations often suggest a strong association between oxidative stress and TGM2 upregulation, which again directs activity and function toward either cell survival or apoptosis. Strong TGM2 upregulation and activity have been reported in some of the most severe neuropathological disorders such as ischemia and diseases such as Alzheimer’s, Parkinson’s, Huntington’s, and amyotrophic lateral sclerosis [22]. Biochemical studies suggest that there may be a link between intracellular reactive oxygen species elevation and glutathione (GSH) depletion and TGM2 upregulation [23], possibly inferring a direct consequence of S-glutathionylation on TGM2. This hypothesis has been further substantiated by Khosla and colleagues in relation to the formation of intramolecular disulfides among a Cys 230-370-371 triad [24], and cysteine residues in TGM2 have been shown to be potentially *S*-glutathione modifiable, with Cys 230 and Cys 370 being related to transamidation activity for their participation in the catalytic triad, while cysteines 524 and 554 may be signaling-related, likely pertaining to TGM2’s duality as a G protein [25].

Functionally, the glutamine transamidation reaction renders the target resultant local structure unrecognizable by proteases to evade proteolysis. In the context of celiac disease, TGM2 pathologically modifies the lining of the small intestine and triggers an autoimmune response against TGM2 and the tissue itself, leading to bowel inflammation, truncated villi, and scalloping [26]; ulcers and bowel obstructions [27]; as well as increased risks of adenocarcinoma of the small intestine and enteropathy-associated T-cell lymphoma. The effect of TGM2 on cell death, however, appears to be dependent on its localization and activation, as during apoptosis, the enzyme migrates from the cytosol to the nucleus to initiate transcription, a behavior that leads to the direct modulation of transcriptional activity in neurodegenerative diseases, likely as a consequence of direct Rb interaction [28] (Figure 2, purple arrows), which lead to Rb to sustain its antiapoptotic transcriptional activity. There are also reports that TGM2 at times undergoes mitochondrial translocation and thus affects ATP synthesis [29,30]. TGM2 is also said to be a critical element in the proper phagocytosis of apoptotic cells [30], although there is no clear correlation between apoptosis and TGM2 expression; for instance, TGM2 overexpression in fibroblasts did not result in increased endogenous rates of cell death in one report [31]. Recently, TGM2 was found to promote migration and invasion in lung cancer cell metastasis, although the involvement was more likely the consequence of translocation rather than transamidase activity [32]. Molecularly, in lung cancer cells TGM2 appears to promote DNA damage repair upon its translocation to the nucleus and interaction with topoisomerase IIα [33], further highlighting that the presence of TGM2 in the nucleus is a trigger for a number of events related to cancer progression. Elevated TGM2 expression has been associated with worsened NSCLC prognosis, namely as a consequence of increased invasion and migration of NSCLC cells [34]. This was manifested in a Korean cohort study of 429 NSCLC patients; a cohort study in China also found associations with shortened survival periods [35]. Additionally, while a relatively large number of potential protein–TGM2 interactions have been characterized [19], few studies have attempted the systematic profiling of TGM2 targets to date.

## 3. Protein Serotonylation

**Figure 3:**
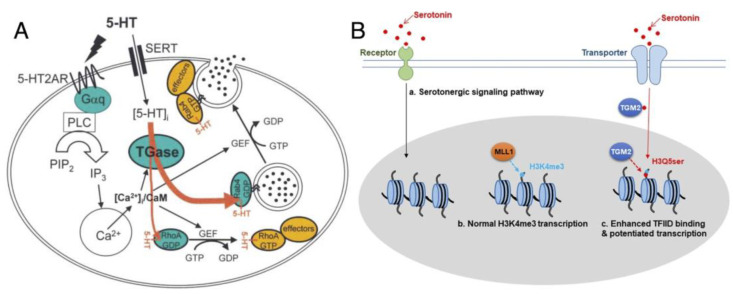
Examples of protein serotonylation: (**A**) Serotonylation of small GTPases. Upon stimulation of platelets by 5-HT, the phosphatidyl inositol pathway is activated, resulting in a rise of cytoplasmic Ca^2+^. 5-HT signaling is mediated by the G-protein-coupled 5-HT2AR and by transport into the cytoplasm by SERT. The contents of α-granules are released into circulation (for example, vWf, black circles) or exposed at the plasma membrane; figure reproduced from [15] (© 2003 Cell Press). (**B**) Regulation model of 5-HT and effect of histone H3 serotonylation, reproduced from Fu et al. [44] (distributed under the terms of the Creative Commons Attribution 4.0 International License, available creativecommons.org/license/by/4.0). The H3K4me3Q5ser double modification enhances the interaction of histone with certain H3K4me3 “reader” proteins, such as TFIID, and then reinforces permissive patterns of gene expression.

There is great diversity in TGM2’s amine donor substrate receptibility when it comes to post-translational protein transamidation, collectively known as “protein monoaminylation”, with different biogenic amines according to their separate involvement in signaling pathways [36]. Among these, serotonylation utilizes 5-hydroxytryptamide (5-HT, serotonin), a vasoconstrictor, as the amine donor to initiate events such as platelet aggregation via the release of von Willebrand (vWf) factors and GTPase activation [15] as reported in 2003 (Figure 3A). This modification rendered serotonylated targets constitutively active, and the effect was primarily attributed to the concomitant activation of G-coupled receptors; at that time, proteins such as vWf, fibrinogen, as well as small GTPases in the range of 20–25 kDa, were also hypothesized to be serotonylation targets.

Later, it was suggested that 5-HT would covalently modify systemic arterial proteins by acting as a substrate for transglutaminase in a model system of rat aorta, forming a complete serotonergic system to control contraction [37]. Filamin A, myosin heavy chain, and actin were identified as targets of serotonylation, although there was yet no site-specific information to ascertain such findings; few to no follow-up studies also surfaced until 2009, when the serotonylation of Rab3a and Rab27a was reported to be a modulatory mechanism in insulin secretion [38]. Other GTPases such as RhoA [39] and Rab4 [40] in muscle cells, as well as Ras [41] in colorectal cancer cells, could also be serotonylated. Other than GTPases, actin and fibronectin were also the recipients of serotonylation [37,42]. Fanburg and colleagues identified serotonylated fibronectin [43] in smooth muscle cells. The modification of fibronectin induced functional responses in the tissue, leading to accumulation that, along with serotonin transporter (SERT) expression, was found to be correlated with the progression of pulmonary hypertension.

However, most of the reports on the phenomenon of protein serotonylation have been relatively focused on signaling; given that transglutaminases remain largely inactive unless stimulated, serotonylation likely serves as a “last line of defense” to force sustained activity onto the important targets involved in signaling. However, the recent reports of histone H3 serotonylation at H3Q5 in H3K4me3-marked nucleosomes by Farrelly et al. [14,44] (Figure 3B) would suggest otherwise, indicating that TGM2-mediated monoaminylation has a larger biological role than what we imagined. This modification leads to the enrichment of euchromatin in serotonergic neurons, by elevating their sensitivity to cellular differentiation and permissive gene expression, achieved by potentiating interactions of TFIID with H3K4me3. H3Q5 is later reported to be modifiable by TGM2 using dopamine as a different amine donor [45]; in this context, the modification can alter cocaine-induced transcriptional plasticity in the midbrain in mice. These new reports provide new glimpses into the role of TGM2 in cancer, although the substantiation of such a claim has been difficult with the scant amount of direct evidence to date.

### 3.1. Proteomic Profiling of Serotonylation Targets

Based on the role of 5-HT signaling, there is speculation that serotonylation may potentially be a supplementary process in the modulation of endocytosis [46]. This belief runs in parallel to the idea that the covalent coupling of neurotransmitters such as 5-HT may prolong the effects of the signaling process, effectively enriching 5-HT local concentration in a phenomenon referred to as the “hormone effect” [36]. Indeed, serotonylation is intimately linked to the presence of transporters such as SERT, since the transporter-mediated uptake of these neurotransmitters from the extracellular space can impact the duration and magnitude of extracellular signaling [47,48,49]. Although for this effect to propagate, it naturally follows that serotonylation targets need to either be highly abundant or sufficiently diverse inside the cell; in order to substantiate the hypothesis of the hormone effect, the complete proteome profiling of serotonylation targets is necessary.

**Figure 4:**
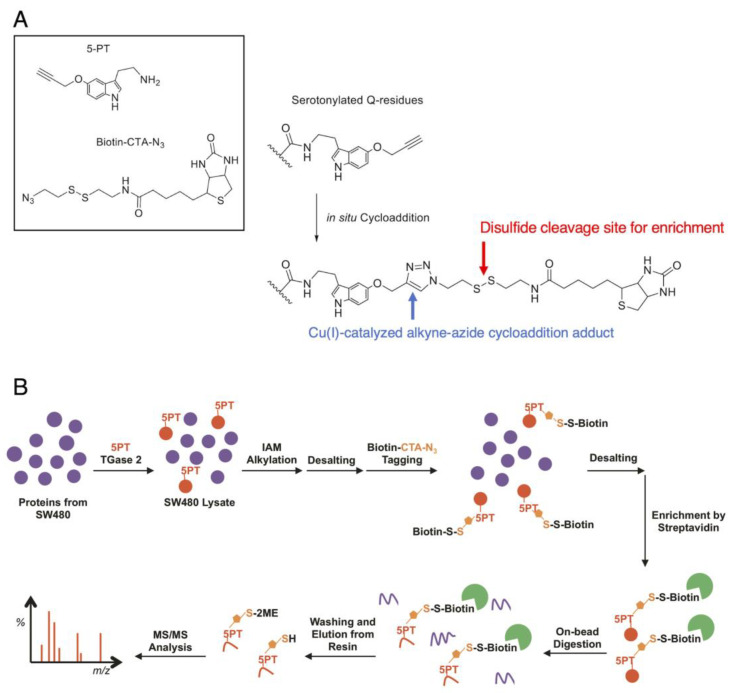
Protein serotonylation by 5-propargylserotonin: (**A**) The approach utilizes the click reaction between 5-propargylserotonin (5-PT) and biotinylazido-cystamine (biotin-CTA-N3, both as shown in the box in the upper left) as a TGM2 surrogate for 5-HT. The conjugation occurs in situ to produce a triazole adduct (right, blue arrow) that is stable under mass spectrometry. When a cleavable moiety is integrated into the linker, e.g., a disulfide structure (right, red arrow), the biotin moiety can be cleaved for elution. (**B**) A proteomic workflow to enrich serotonylated proteins.

The road to the comprehensive proteomic profiling of serotonylation targets, however, still remains a difficult technical challenge. The ubiquitous and wide availability of biogenic monoamines in nature often leads to the generation of ineffective antibodies, most of which are relied upon for the enrichment of transamidated proteins in vivo. While serotonylation has been characterized with isotopic variants of 5-HT such as ^14^C-5HT [15] and ^3^H-5HT [38], radioimaging methods are not readily transferrable to larger-scale profiling. There have also been attempts with biotin-conjugated 5-HT to probe serotonylation [37], although there is a possibility that the increased size of the biotinylated adduct can affect TGM2 activity. We originally devised a Cu(I)-catalyzed alkyne–azide cycloaddition -based method (Figure 4A) to generate a derivative of 5-HT much comparable in size to 5-HT. Using this construct, we characterized more than 50 modification sites in 46 proteins from mass-spectrometric proteome profiling in SW480 colorectal cancer cells [50] (Figure 4B), in what was perhaps the first report of serotonylation proteomic profiling. The proteins belonging or closely associated with the heat-shock protein (HSP) family, folding, and structural assembly were identified as serotonylation targets, including PSMD9 (a chaperon for 26S proteasome assembly), heat-shock proteins such as DNJB1 (Hsp40) and GRP78 (HspA5), as well as radixin, RanBP-1, etc. All of these elements have varying degrees of interactions with the elements related to Akt signaling and subsequently lung cancer. While there was no conclusive serotonylation motif from the profiling results, modification tended to occur near a relatively higher abundance of aliphatic, non-polar amino acids in the immediate vicinity; this echoed the earlier observations of TGM2 preferring glutamines in proline-rich regions [51]. Farrelly et al. later also employed this approach to investigate histone serotonylation in HeLa cells [14].

### 3.2. The Abundance Hypothesis

There is sufficient evidence to declare that TGM2 is linked to a number of diseases and also precancerous conditions and that it activates caspase 3 and possibly crosslinks proteins in a fashion that propagates irreversible amyloid formation. It is even understood that TGM2 readily accepts chemically modified polyamines as substrates of transamidation and prefers the functional group adduct in a linear fashion rather than branched [52]. However, we still know very little about the rationale behind the choice of biogenic monoamines as substrates of TGM2. There is a common belief—for the sake of discussion, we shall refer to this as “the abundance hypothesis”—that TGM2 will non-preferentially utilize freely available amines as donors as necessary by the virtue of its primary biological role: to evade autoproteolysis and apoptosis acutely, where substrate preference may hinder the rate of this very function. Since different proteins can have varying degrees of amine donor acceptancy, TGM2 may reserve priority and exert preference in selecting a particular biogenic amine as its substrate for modification; to override such this preference, great excess quantities of a particular amine near 10 mM are often required to achieve incorporation [52,53,54]. As the phenomenon of serotonylation has been observed in organs other than the gastrointestinal tract, we can also safely presume that there is a specific and deliberate rationale that TGM2 may preferentially seek to transamidate proteins with 5-HT, rather than other available and nearby biogenic monoamines. Another fallacy with such a hypothesis is that these amine donors often require complex pathways of biosynthesis, most of which can be energetically expensive and have drastically different yields. Spermine is not biologically equivalent to 5-HT or dopamine, despite the fact that nearly all of them are regularly utilized as amine donors by TGM2. Since nearly 95% of 5-HT is accumulated in the gastrointestinal tract, with the vast majority found in enterochromaffin cells and the remaining 10% or so in enteric neurons [55], the mere fact that a nominally small quantity of 5-HT is found outside these systems implies that the trafficking of serotonin to these systems must be purposeful as a consequence of the tightly regulated and energetically costly biosynthetic process.

## 4. Implications of Protein Serotonylation in Oncogenesis

Over the decade since Walther and colleagues first reported the phenomenon [15], little remained known about the connections between serotonylation and its connections to diseases such as cancer. The serotonylation of small GTPases, while serving a signaling function, is unlikely to be a facilitating factor in oncogenesis due to their rapid degradation, likely proteasomally [39], soon after they become active, not to mention the relatively short livelihood of TGM2 itself [20]. Other factors such as TGM2’s activation of caspase 3 and its involvement in redox imbalance [56] are thus more likely to be the key player in the process instead. Reports so far do seem to suggest that serotonylation can exert epigenetic effects in modulating the relaxation of condensed chromatin to permit transcription. Furthermore, proteomic profiling has revealed a number of heat-shock proteins and chaperons, such as PSMD9 and DNAJB1 (Hsp40), to be serotonylation targets [50]. CHMP4B is essential for BMP4-mediated chromosomal integrity safeguarding, a process that is linked to the increased levels of H3K4me3 modification in the CHMP4B promoter [57]. DNAJB1 in particular has been said to interact with STUB1 (also known as CHIP) and HSPA4 [58,59]; alteration in the DNAJB1 interaction with STUB1 by the virtue of serotonylation is likely to affect MLL5, an element said to be intimately related to H3K4 transferase activity [60]. GRP78 may also promote tumor cell histone acetylation [61]. The serotonylation of these protein targets may subsequently affect histone modification and subsequently the epigenetic landscape through a number of other elements such as other PTMs, transcription factors, or galectins that compete for or allosterically hinder access to DNA.

With TGM2’s possible interactions with HSPs such as Hsp70, Hsp20, and Hsp27 having been demonstrated on a cellular level [62,63,64], HSPs may also be targeted for sustained activation to alleviate stresses exerted during periods of distributed homeostasis and possible apoptosis, as triggered by the influx of serotonin. As HSPs are said to be exploitable targets for cancer immunotherapy [65,66,67], the act of triggering a large expression increase in cancer cell HSPs by serotonylation may assist in sensitizing therapeutic regimens. Aside from HSPs, another connection of note is the link to the epigenetic landscape via chromatin modifiers and histone modifications. Relations to cancer immune response can be gleaned from CD34, another target of protein serotonylation at Gln 377. Unlike most cancers, SCLC can in fact express CD34 [68]; it follows that alteration in the CD34 state through serotonylation may potentially alter hypermethylation patterns in promoter CpG islands in acute myeloid leukemia [69]. Since galectins, e.g., galectin-3, are often co-expressed with CD34 in cancers such as hepatocellular carcinoma [70] and can contribute to the tumor’s ability for immune invasion by increasing matrix metalloproteinase activities [71], there may be direct associations that merit further investigation, especially given the fact that serotonylation may increase PD-L1 expression [72]. In the context of treatment for lung cancer, given the links between histone modifications and permissive reprogramming as a consequence of chromatin structural changes, it is possible that HDIs such as vorinostat may reverse the effects of certain histone modification roadblock events such as DOT1L-mediated H3K79/K27 methylation [73]. There may also be connections to protein serotonylation through aspects of cancer immunotherapy via alterations in CD34 structural modifications through serotonylation.

## 5. Associations of Protein Serotonylation with Other Epigenetic Features

### 5.1. Implications of Histone Modifications in Cancer

Transcriptional regulation usually occurs through a series of modifications or changes in histone elements to prevent the physical process of transcription from being carried out successfully. Epigenetic changes such as microRNA, CpG island sRNA, and nucleosome positioning all have different roles in transcriptional regulation; for instance, the POU (Pit-Oct-Unc) transcription factor family stimulates the transcription of their target genes by interacting with basal transcriptional mechanisms and a number of other factors [74,75]. In cancer cells, however, genomic aberrations may easily tip the delicate balances and cause misguided activation of oncogenic drivers, and through the same mysterious act conversely deactivate tumor suppressors. In this review, for the sake of discussion we will narrow the context of genomic aberrations to only histone modifications.

Histones are the central component of the nucleosomal subunit, forming an octamer containing core histone proteins, namely H2A, H2B, H3, and H4 [76]. Notably, there is established evidence of TGM2 interaction with Histone 2B [19]. Changes in PTM patterns of histone are extensively linked to cancer, both at the genome level and specific loci [77,78]. On the other hand, some of the most frequently mutated targets in cancers have also turned out to be mutations in histone-related elements as well [79]. A number of PTMs can occur in histone, such as acetylation, methylation, phosphorylation, and adenosine diphosphate (ADP) ribosylation. On the other hand, ADP-ribosylation by NAD+-dependent poly(ADP-ribose) polymerases (PARPs) and class III HDACs such as Sirtuin [80] histone is a consequence of PARP1 becoming activated due to environmental stress, causing local DNA damage. PARP1 modifies the amino-terminal tails of core histones, for instance, at residues H2AK13, H2BK30, H3K27, H3K37, and H4K16 [81], and leads to chromatin decondensation and the recruitment of DNA repair machinery. PARP1 inhibitors have been shown to counteract the effects of these modifications in SCLC [82], not to mention as treatment of breast and ovarian cancers. Other histone PTMs, such as citrullination, ubiquitination, formylation, *O*-GlcNAcylation, lactylation, propionylation, butyrylation, crotonylation, benzoylation, and proline isomerization, can occur near the basic and flexible histone tails [83,84,85,86,87,88,89,90,91,92]. Citrullination modifies histone arginine residues as a consequence of methylation by protein arginine methyltransferases; in effect, the positive charge on arginine is removed enzymatically by the partitioning and anchoring domain proteins (PADs) [93]. Particularly, PAD4 has a nuclear localization signal and is reported to modify histones H2A and others, i.e., at residues H1R5, H3R2, H3R8, H3R17, and H3R26 and H4R3 [94,95,96,97], leading to chromatin decondensation [96,98]. PAD4 is also highly expressed in a number of cancers, for instance, SCLC, ovarian, breast, and hepatocellular carcinomas [99]. We should also note that these modifications have different yet concerted regulatory effects on gene expression; one of the most famous is perhaps acetylation and methylation in their ability to activate or deactivate transcription. Aside from methylation repressing histone acetylation, multiple methylation states can exist at the same lysine or arginine residue, adding to the complexity of the interplay. The methylation/acetylation pair is notably regulated by multiple methyltransferase writers and demethylase erasers to dictate changes in cell fate and genomic stability [100]. Of course, we cannot discount recent discoveries that serotonylation and dopaminylation can also modify histones, with severe implications in neuron differentiation and cocaine addiction [14,45]. The dopaminylation of histone H3 has an impact on ventral tegmental area function and, consequently, on dopaminergic action potentials. The result is aberrant dopamine signaling in the ventral striatum during the periods of drug seeking [45]. H3Q5ser, when coupled with H3K4 methylation, can augment the potentiation of chromatin readers and erasers, thus highlighting the possibility of serotonylation being a hidden third man in the acetylation–methylation concert pair. For instance, H3K4me3 readers have been said to be able to tolerate H3Q5ser while H3Q5ser can stimulate KDM5B activity with little to no effect on the binding kinetics of H3K4me3 readers [101]. While these modifications can be used as therapeutic targets, we need to be particularly aware of their concerted efforts to modulate the epigenetic landscape. Even though a number of publications have alluded to such concerted crosstalks, most remain merely on-the-surface citations to H3 modifications; thus, a more thorough investigation into this subject matter will be necessary in the future.

### 5.2. Implication of Galectins and Their Relationships to Histone H3 Modifications

HDAC inhibition may also lead to alterations in the glycome, as evident in the rise in a number of differently expressed glycosidases such as TPTST2, NEU1, FUCA1, GALM, etc. [102], again hinting at the complex interplay between PTMs in the modulation of the epigenetic landscape. For instance, aldolase A, an interacting partner of TGM2 [19], has been found to promote lung cancer metastasis, likely via its interaction with actin [103] and the subsequent alteration in histone–actin interactions [104]. While chemical biology-based tools such as enrichment-based proteomics or activity-based probe profiling, such as those based on alpha-fluoromethylphenyl fucopyranoside reaction intermediates for α-*L*-fucosidase [105], can help establish clear and direct relationships, so far there appear to be no reports that utilize such methodology. Furthermore, as the relationship between galectins and cancer has long been investigated, these sugar binders are also likely to have a hand in the epigenetic modulation connected to TGM2-mediated protein serotonylation. Galectins are an ancient and evolutionarily conserved protein family that recognize β-galactose-containing glycoconjugates, and they have a diverse range of functions relevant to a wide variety of biological events and diseases, including developmental processes, cell migration, immune regulation, antimicrobial activity, apoptosis, and cancer [106,107,108,109,110]. The presence of galectins in various cellular components, including extracellular and intracellular spaces, and changes in galectin expression are believed to be critical for cancer progression, metastasis, and angiogenesis, especially in relation to their involvement in a number of different signaling pathways [111]. Notably, histone modification leading to epigenetic changes can regulate galectin activity in cancer. Galectin-9, as an example, has been noted to have its expression levels linked to CpG methylation, as well as H3K9 and H3K14 histone acetylation near the galectin-9 promoter [112]. Similarly, galectin-1 has also been found to have moderate expression gains in mixed lineage leukemia (MLL)-rearranged B-lymphoblastic leukemias, with evidence of hypermethylation related to H3K79 in its promoter region [113]; H3K79 dimethylation is also connected to changes in the histone methyltransferase activity of the MLL fusion protein complex [114]. Even though the impact of epigenetic modulation on galectin expression is imperative, there is limited information at this time on their direct connections to histone modifications and epigenetic changes that regulate galectin activity in cancer.

## 6. Conclusions

Over the past two decades, from the first report of GTPase being targets of serotonylation in 2003 to the latest references on histone H3 modification, our understanding of protein serotonylation still remains an unsolved puzzle on a coffee table in the corner of the living room, just right out of sight. Unlike other modifications, there is still no “one-size-fits-all” functional definition for serotonylation. we know that this PTM likely affects signaling, but the exact extent of “how” remains largely unsubstantiated without more comprehensive profiling of the proteome. Likewise, while we can definitively affirm that the serotonylation of H3Q5 can affect the epigenetic landscape, questions such as the reason for the presence of serotonin in the nucleus, or whether there exist other concerted PTM pairings to assist epigenetic modulation, still linger and muddle the discussion. The presence of serotonin in the mitochondrion and the activation of serotonin receptors in intracellular membranes [115] do indeed align with our knowledge of TGM2 distribution and its occasional role as a G protein; following this logic, it may be safe to presume that serotonylation is a conduit for TGM2 to perform a signaling function that may otherwise be served with other GTPases. Under this hypothesis, TGM2’s role as a G protein may then explain the occurrence of H3 serotonylation when we take into consideration the role of G-protein-coupled receptors, which can act as an HDAC kinase [116]. While this may be the missing puzzle piece that we have misplaced all along, it is imprudent to jump to conclusions without a more thorough examination of other elements at play such as interactions with galectins, receptor elements, and other histone PTMs. Alas, the detailed profiling of serotonylation targets as well as a mechanistic investigation of the process of de-serotonylation are also important topics that remain unaddressed even to this day.

## 7. Future Directions

In the context of designing therapeutic options for lung cancer, a good starting point is the optimization of HDIs such as vorinostat. Unmodified vorinostat is a relatively broad spectrum and can cause a large number of undesirable adverse effects, as do most HDIs; one pressing issue to alleviate some of these side effects is to optimize the drug to allow more specific target homing. This aspect of HDI optimization is particularly important even in combination therapies since most other combination drugs are often also broad-spectrum cytotoxic agents. A possibility is the use of minor-groove binding molecules such as *N*-methylpyrrole-*N*-methylimidazole polyamides (PIPs) to isolate vorinostat to only certain genomic targets. An example of this is SAHA-PIPs, which were shown to be capable of selectively upregulating certain genes related to chromatin reprogramming and subsequently inducing pluripotency in mouse embryonic fibroblasts [117]. SAHA-PIPs may have great implications as oncotherapeutic candidates, as their tradeoff of a narrower range of genomic targets compared with vorinostat may translate to heightened effects for their targets and fewer undesired adverse effects as a consequence of reduced off-target binding [118], in which more systematic investigations may be required. It may also be worthwhile to examine how HDIs interact with the transcriptional and epigenetic machinery to optimize the engineering of a new generation of HDIs for cancer therapy. With the advent of artificial intelligence, it may be possible to utilize modeling tools such as AlphaFold [119] to simulate conformational changes to some of the machinery. For example, changes in conformation upon binding with SAHA-PIP/DNA ligand or changes in surface accessibility of H3Q5 via AlphaFill [120], a derivative of AlphaFold with ligand-binding capabilities, may help understand how histone deacetylase interacts a ligand of SAHA-PIP with methylated DNA to achieve selective activation of tumor suppressor genes. While at this time we are no longer actively investigating the phenomenon of protein serotonylations directly, we do believe that a more thorough examination of these histone elements at play, such as interactions with galectins, receptor elements, and other histone PTMs, is a necessity moving forward, along with systematic profiling of serotonylation targets in the cancer proteome to provide a more comprehensive picture to understand the role of this uncommon PTM.

## Appendix A

The five-year survival rates for both NSCLC and SCLC were estimated from the number of cases diagnosed between 1997 and 2014 from participating member hospitals in the national cancer registry of Japan. Survival rates for NSCLC were estimated based on the records categorized as large-cell carcinoma across all clinical stages (*N* = 1889, 83.2% men; mean age = 65.1 with a standard deviation of 10.6). SCLC values were based on the records cataloged as SCLC and large-cell type neuroendocrine carcinoma and included a total of 8827 cases, 83.8% of which involved men, across all stages of the disease, with a mean age of 68 and standard deviation of 8.9. Calculations were performed with KapWeb [121], with the database last updated in November of 2022.

## Appendix B

The claim that vorinostat “as is” (i.e., not chemically derivatized) has failed to show efficacy for lung cancer is based on the results available from ClinicalTrials.gov, in which “vorinostat” and “lung” were used as search keywords. At the time of writing, 32 clinical studies had been cataloged, 14 of which were terminated or completed studies with publicly viewable results. Notably, 3 of the 14 studies had enrollment greater than 30 subjects (NCT00481078, *N* = 94; NCT00473889, *N* = 253; and NCT00423449, *N* = 61). None of the aforementioned studies provided sufficient evidence to support the hypothesis that unmodified vorinostat in combination therapies performed statistically better than the placebo in the case of lung cancer. As no other posted results included comparative studies of vorinostat vs. placebo alone, there was also no supporting evidence that the combination therapies of unmodified vorinostat with other drugs such as paclitaxel or carboplatin increased efficacy against lung cancer.
